# M. Begin on the Treatment of Hæmorrhage after Lithotomy

**Published:** 1842-05-07

**Authors:** 


*Hæmorrhage after Lithotomy.*—At the Academy of Medicine, Paris, M. Begin read a memoir on the hæmorrhage which occurs after the operation of lithotomy. If we consider the normal anatomy only (says the author) of the parts concerned in the operation of lithotomy, we shall have a very imperfect idea of the cause and frequency of this accident. In many cases the occurrence of hæmorrhage depends on various anomalies in the course and volume of the vessels, and on their dilatation; but in others the bleeding takes place by a sort of exhalation, without the injury of any considerable vessel.

The statistics of lithotomy show, that one out of every five or six patients, who are cut for the stone, die; it would be highly useful if we would determine in what proportion the different accidents that attend the operation contribute to this mortality. This is a very difficult question, and all that the author attempts to do is, to affirm generally that about one-fourth of the total deaths depend upon hæmorrhage. Authors lay much stress on the necessity of determining the exact seat of the bleeding, and they give several rules on this point; but M. Begin observes, that it is always very difficult to form any precise idea of the vessel from which the bleeding comes. The author next passes to an examination of the various means employed to arrest the hæmorrhage—viz. pressure, ligature, plugging, cauterisation, cold injections, &c. &c.; he affirms that none of these means can be confidently relied on in all cases. He then mentions a process which he has adopted with success in three cases where the bleeding had resisted every other means. In the first case he had the patient placed on the edge of a bed, and made his pupils keep constantly injecting cold water into the wound. After the lapse of an hour the bleeding ceased, and did not return. This method, however, is extremely tedious and difficult of execution; the author, therefore, in a second case, substituted for it the keeping up a constant current of fluid, by means of a tube placed in a pail of cold water.—*Provincial Journal.* March 5th, 1842.

